# Reduced Expression of the Extracellular Calcium-Sensing Receptor (CaSR) Is Associated with Activation of the Renin-Angiotensin System (RAS) to Promote Vascular Remodeling in the Pathogenesis of Essential Hypertension

**DOI:** 10.1371/journal.pone.0157456

**Published:** 2016-07-08

**Authors:** Yuan-yuan Qu, Jing Hui, La-mei Wang, Na Tang, Hua Zhong, Yong-min Liu, Zhen Li, Qian Feng, Fang He

**Affiliations:** 1 Department of Pathophysiology/Key Laboratory of Education Ministry of Xinjiang Endemic and Ethnic Diseases, Medical College of Shihezi University, Shihezi, China; 2 Department of Emergency and critical care medicine, the First Affiliated Hospital of Medical College of Shihezi University, Shihezi, 832008, China; 3 Centre of Medical Functional Experiments, Medical College of Shihezi University, Shihezi, China; Universidade Federal do Rio de Janeiro, BRAZIL

## Abstract

The proliferation of vascular smooth muscle cells (VSMCs), remodeling of the vasculature, and the renin-angiotensin system (RAS) play important roles in the development of essential hypertension (EH), which is defined as high blood pressure (BP) in which secondary causes, such as renovascular disease, are absent. The calcium-sensing receptor (CaSR) is involved in the regulation of BP. However, the underlying mechanisms by which the CaSR regulates BP are poorly understood. In the present study, the role of the CaSR in EH was investigated using male spontaneously hypertensive rats (SHRs) and rat and human plasma samples. The percentages of medial wall thickness to external diameter (WT%), total vessel wall cross-sectional area to the total area (WA%) of thoracic arteries, as well as the percentage of wall area occupied by collagen to total vessel wall area (CA%) were determined. Tissue protein expression and plasma concentrations of the CaSR, cyclic adenosine monophosphate (cAMP), renin, and angiotensin II (Ang II) were additionally assessed. WT%, WA%, and CA% were found to increase with increasing BP, whereas the plasma concentration of CaSR was found to decrease. With increasing BP, the levels of smooth muscle actin and calponin decreased, whereas those of osteopontin and proliferating cell nuclear antigen increased. The CaSR level negatively correlated with the levels of cAMP and Ang II, but positively correlated with those of renin. Our data suggest that reduced expression of the CaSR is correlated with activation of the RAS, which induces increased vascular remodeling and VSMC proliferation, and thereby associated with EH in the SHR model and in the Han Chinese population. Our findings provide new insights into the pathogenesis of EH.

## Introduction

The extracellular calcium-sensing receptor (CaSR) belongs to family C of the G-protein-coupled receptors, which are also known as seven transmembrane domain receptors. The CaSR is expressed in all major organs involved in Ca^2+^ homeostasis, including the parathyroid gland, kidney, and bone[1, 2]. Furthermore, increasing evidence suggests that the CaSR, which senses changes in extracellular calcium concentrations ([Ca^2+^]_o_), is expressed functionally in the outer membrane of the blood vessel wall, fibroblast cells, vascular smooth muscle cells (VSMCs)[3–5]and endothelial cells[6]. Numerous studies have shown that low levels of dietary Ca^2+^ represent a significant risk factor for hypertension, while the intake of appropriate amounts of Ca^2+^ effectively lowers the BP[7]; this has been confirmed by studies in animals[8]. As early as in 1911, scholars such as Cow found that elevated Ca^2+^_o_ concentration elicits a vascular relaxation response in vitro[9]. Increased [Ca^2+^]_o_ induces the binding of Ca^2+^ to the CaSR and activates the G-protein-phospholipase C(PLC)-inositol 1,4,5-trisphophate (IP3) receptor pathway, triggering an elevation in intracellular Ca^2+^ concentrations ([Ca^2+^]_i_), which is implicated in the development of cardiovascular diseases such as hypertensive disorders. Ogata et al. [10]reported that NPSR-568 (R-568), an allosteric activator of the CaSR, reduces the blood pressure (BP) in uremic rats and spontaneously hypertensive rats (SHRs), but has no effect on normotensive rats. Rybczynska et al. [11, 12]reported that NPS 2143, an allosteric inhibitor of the CaSR, increased BP in normotensive rats; however, this hypertensive effect was not observed in rats with surgically removed parathyroid glands, or in the presence of calcium channel blockers or antagonists of angiotensin II (Ang II) type 1 (AT1R) receptors (e.g. losartan). The biological mechanisms underlying these effects are not completely understood.

Essential hypertension (EH), which is defined as elevated blood pressure in which secondary causes, such as renal failure, are absent, is a serious disorder that results in damage to blood vessel walls and is associated with a higher risk of stroke. EH is a complex disorder resulting from both genetic and environmental factors[13]. The renin-angiotensin system (RAS) plays an important role in the development of EH. Renin, which is closely related with hypertension, is the first rate-limiting enzyme of the RAS, and cAMP functions as a key effecter in this system[14]. EH may be classified into three clinical types based on the level of renin: high-, normal-, and low-renin types. About 25%-33% of EH patients exhibit low renin levels; accordingly, the majority of hypertensive patients in China suffer from low-renin hypertension (LRH), a well-known subtype of EH[15]. LRH, which is related to excess sodium retention and concomitantly increased extracellular fluid volume, shows a causal relationship with hypertension. Davies E et al. [16] found that polymorphisms in genes associated with the RAS, such as the T344C polymorphism of the CYP11B2 gene, may be involved in the occurrence of LRH. In addition, Boucher described a novel enzyme, tonin, that catalyzes the formation of Ang II directly from a plasma protein using the renin tetradecapeptide substrate and angiotensin I, which are present in most tissues[17]. Ang II is a peptide hormone that mediates vasoconstriction and increases BP by binding to its corresponding type 1 receptors (AT1R). Ang II therefore plays an extremely important role in hypertension and related complications[18].

In vitro and in vivo, renin-secreting juxtaglomerular (JG)cells express the CaSR, and the activation of the CaSR with a calcimimetic has been shown to inhibit the release of renin [7]. Ortiz-Capisano et al. reported that activation of the CaSR activates the ryanodine receptor (RyR) via the PLC/IP3 pathway, thereby increasing [Ca^2+^]_i_, suppressing cAMP formation, and inhibiting renin release in primary cultures of isolated mouse JG cells[19].

Our previous studies have shown that the CaSR plays an important role in spermine (a CaSR agonist)-evoked Ca^2+^ influx and nitric oxide production in human umbilical vein endothelial cells (HUVECs), indicating a role for the CaSR in regulating BP[20]. However, little is known about the role of the CaSR in phenotypic modulation of VSMCs in hypertension, and the underlying mechanisms are poorly understood. We hypothesize that CaSR expression and/or functional impairment weakens the inhibition of adenylate cyclase (AC) and increases cAMP formation and renin release, resulting in activation of the RAS, which induces VSMC proliferation and remodeling, thereby promoting the development of hypertension. In the present study, the correlation between the expression of CaSR, vascular proliferation and remodeling, and the development of EH, as well as the underlying mechanisms, were investigated. Our findings suggest that reduced CaSR expression is associated with activation of the RAS, which promotes vascular remodeling, thereby playing an important role in the development of EH. These results suggest that the vascular CaSR pathway may be a therapeutic target in the management of hypertension.

## Materials and Methods

Study protocol was approved by the ethics committee of Medical College of Shihezi University, and all participants provided written informed consent. The participants also provided written consent for their blood samples to be retained for future research. The animal study was approved by the Institutional Animal Research Committee of Shihezi Medical University, and all animals received humane care in compliance with the Guide for the Care and Use of Laboratory Animals published by the National Institutes of Health (NIH Publication 86–23, revised 1986).

### Patient characteristics

We used the 1999 WHO/ISH Hypertension Guidelines for diagnosing hypertension, which is defined as systolic BP (SBP) ≥140 mmHg (18.7 kPa) and/or diastolic BP (DBP) ≥90 mmHg (12.0 kPa). Data as well as samples were collected as previously described[21]. Briefly, BP was measured after the participants had rested for 15 minutes, and the measurement was repeated three times. During the investigation, overnight fasting blood (10 mL) was drawn and processed (centrifuged, separated, frozen, and packaged in a −80°C freezer). In our previous case-controlled study, total 2300 participants were included in the study. From this pool, we choose 200 Han Chinese Population (age: 18–84 years) in the investigation (106 hypertension patients, 56 males and 50 females; and 94 healthy participants, 50 males and 44 females), using random cluster sampling, gender and age-matched. People taking drugs for the treatment of hypertension or with heart disease or renal or en docrinological diseases that cause secondary hypertension were excluded from this study.

### Animal protocol

Twenty one male SHRs and the same numbers of WKY rats, aged 8–16 weeks and weighing 200–300 g, were purchased from Vital River Laboratory Animal Science and Technology Co., Ltd, Beijing. License Number: SCXK2012-0001. The SHRs and WKY rats were divided into six groups according to the age of the week (n = 7/group): 8-week-old SHRs group (SHR8w), 12-week-old SHRs group (SHR12w), 16-week-old SHRs group (SHR16w) and age-matched WKY rats groups: 8-week-old Wistar-Kyoto rats group (WKY8w), 12-week-old Wistar-Kyoto rats group (WKY12w), 16-week-old Wistar-Kyoto rats group (WKY16w). All animals were kept under identical conditions of temperature and humidity, and had access to food and drinking water ad libitum.

### Evaluation of EH

Before the experiment, the BP was measured once a day, last seven days, with the tail cuff method, which included warming the whole animal body in the absence of anesthesia (BP-98A-L, Softron, Tokyo, Japan) [22]. Measurements were taken at the same time of day on each occasion at a controlled temperature of 30°C. After the rats were adapted to the environment and detected the stimulus, measure the SBP, DBP and mean arterial pressure(MAP) [MAP = DBP+1/3(SBP-DBP)], measurement was repeated three times, take the average of three times. Subsequently, anesthesia was induced in rats with 10% chloral hydrate. Blood was withdrawn from the abdominal aorta, plasma samples were obtained by blood centrifugation and stored at -80°C. Rat thoracic aorta was isolated and then cut about 0.5 cm, placed in 10% neutral buffered formalin for histopathological evaluation [22]. The remaining parts were snapped frozen in liquid nitrogen until measurements of tissue were performed.

### Histology and thoracic aortic vascular morphology

After fixation, the tissues were embedded in paraffin, five-micron-thick tissue sections were cut from the paraffin blocks. Then the thoracic aortic specimens of rats were stained using hematoxylin and eosin (HE) and Masson trichrome, respectively. To ensure the accuracy and reliability of the results, each section was viewed, measured and evaluated by two blinded investigators. Then, we randomly selected three fields in each slice, calculated the parameters of thoracic aorta vascular cross sections by measuring the wall thickness, cross-sectional vessel area and wall area, total vessel wall thickness, and the percentages of medial wall thickness to the external diameter (WT %), cross-sectional total vessel wall area to the total area (WA %) and collagen area to total vessel wall area (CA%). /The images of the arteries were captured by OLYMPUS BX40 microscope and analyzed using Image-Pro Plus 6.0 software (Media Cybernetics Inc, Buckinghamshire, UK).

### Western blotting analysis

The vascular media of thoracic aorta tissue of every rat was homogenized in lysis buffer and centrifuged at 14000 × *g* for 15 minutes at 4°C, then the supernatant was collected. Electrophoresis was performed on 10% SDS-polyacrylamide gels to transfer proteins to 0.45 mm Sequi-Blot PVDF membranes (23 V, 55 minutes). After incubation with 5% non-fat milk, then incubated overnight at 4°C with primary antibodies against CaSR (1:800, Abcam, Massachusetts, USA), proliferating cell nuclear antigen (PCNA, 1:400, Boster, Wuhan, China), smooth muscle a-actin (SMAa) (1:400, Boster, Wuhan, China), calponin (1:200, Boster, Wuhan, China), osteopontin (OPN) (1:200, Boster, Wuhan, China). Monoclonal anti-β-actin (1:1000, Cell Signaling Technology, Danvers, MA, USA) antibodies were used to standardize each line and as an integral loading control. After being washed, the membranes were incubated with horseradish peroxides (HRP)-conjugated species-specific secondary antibodies (1:25000, Boster, Wuhan, China). Protein was visualized using an enhanced chemiluminescence system (ECL, Pierce Company, USA). Intensities of the protein bands were quantified using a Bio-Rad Quantity One software (Bio-Rad, Hercules, CA, USA).

### Immunohistochemistry analysis

The tissue sections were deparaffinized, rehydrated, washed, immersed in 3% hydrogen peroxide, and then were boiled in 0.01 M sodium citrate buffer (pH = 6.0) in a pressure cooker for 4 minutes for antigen retrieval. The sections were incubated with primary rabbit polyclonal antibodies against CaSR (1:50, Abcam, Massachusetts, USA), OPN (1:25, Boster, Wuhan, China) calponin (1:800, Boster, Wuhan, China) and primary mouse monoclonal antibody against PCNA (1:200, Boster, Wuhan, China) and SMAa (1:400, Boster, Wuhan, China) at 4°C overnight, respectively. Then, slides were washed 3 times in PBS (pH 7.4–7.6) and incubated for 30 minutes with corresponding secondary horseradish-peroxidase-conjugated antibody (Invitrogen, Beijing, China). Diaminobenzidine / peroxidase substrate was used to produce a brown-colored signal. The sections were counterstained, dehydrated, cleared, and coverslipped. Negative controls were performed with the omission of the primary antibody. For quantitative determination of the protein levels, the positive staining in tissue sections was analyzed by integrated optical density (IOD)/vessel wall area using the Image-Pro Plus 6.0 software (Media Cybernetics Inc, Buckinghamshire, UK) as described previously [23]. All images were taken using the same microscope and camera sets.

### Determination of RAS, cAMP and CaSR contents in plasma by ELISA

Treatment and collection of plasma samples as mentioned in the previous, plasma levels of CaSR, cAMP, renin and Ang II (Ang II)were determined by using standard commercially available enzyme immunoassay for the in vitro quantitative measurement in rats and human (USCN Life Science, Wuhan, China), intra-assay and inter-assay coefficients of variation less than 8%. The absorbance at 450 nm was read using a Microplate Reader (Bio-RAD Model 3550-UV, USA).

### Statistical analysis

Quantitative data are presented as mean±standard deviation and were compared among multiple experimental groups using analysis of Student’s *t’* test, One Way Analysis of Variance (ANOVA) or Kruskal–Wallis test, then Bonferroni post hoc tests were used after ANOVA., Linear regression analysis was used to determine correlation between CaSR, BP and other variables. *P*-values less than 0.05 were considered statistically significant. Statistical analysis was performed using the SPSS 17.0 software (SPSS Inc., Chicago, IL, USA).

## Results

### BP changes in SHRs and hypertensive patients

**Fig 1:**
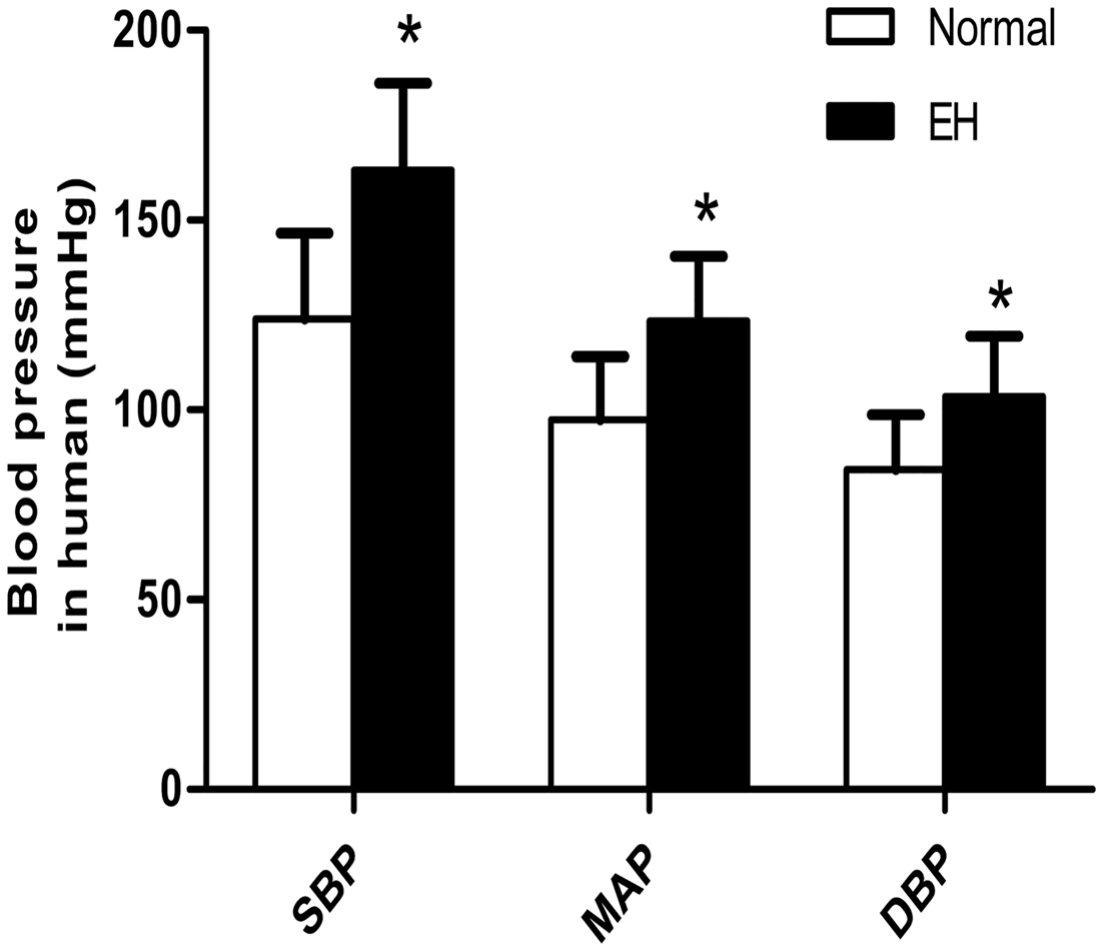
Comparison of systolic blood pressure (SBP), diastolic blood pressure (DBP), and mean arterial blood pressure (MAP) between different groups of humans. **a** Data are presented as means ± standard deviation;*P < 0.05 EH group versus normal group.

BP (including SBP, DBP, and MAP) was significantly higher in the SHRs groups compared with the age-matched WKY rat groups (*P* < 0.05). Additionally, we found that BP and weight of the 16-week-old group was significantly higher than that of the 8-week-old group (*P* < 0.05) (Table 1). There was no difference between the WKY rat groups in terms of BP, and no difference between SHRs groups and the age-matched WKY rat groups in terms of weight (*P* > 0.05). In humans, the BP of the hypertension group was higher than that of the normal group (see S1 Table for data) (*P* < 0.05) (Fig 1).

**Table 1:** Comparison of systolic blood pressure (SBP), diastolic blood pressure (DBP), mean arterial blood pressure (MAP), and weight between different groups of rats.

Groups	SBP (mm Hg)	DBP (mm Hg)	MAP (mm Hg)	Weight (g)
**WKY8w**	131.14 ± 11.38	84.57 ± 12.95	100.14 ± 11.75	211.53 ± 9.80
**SHR8w**	173.43 ± 9.25*	126.86 ± 10.27*	142.43 ± 7.46*	201.43 ± 7.50
**WKY12w**	136.86 ± 10.84	84.86 ± 9.99	102.19 ± 10.15	271.35 ± 7.37
**SHR12w**	182.13 ± 14.96*	138.29 ± 10.26*	152.71 ± 10.11*	269.87 ± 9.76
**WKY16w**	146.22 ± 6.73	99.26 ± 6.47	118.19 ± 5.93	295.62 ± 10.55^a^
**SHR16w**	189.43 ± 13.56*^#^	146.86 ± 8.82*^#^	161.14 ± 8.05*^#^	293.43 ± 12.26^#^

Data are presented as means ± standard deviation;

*P < 0.05. SHRs groups versus the age-matched WKY groups;

^#^*P* < 0.05, 16-week-old SHRs (SHR16w) group versus 8-week-old SHRs group (SHR8w);

^a^*P* < 0.05 16-week-old WKY rats (WKY16w) group versus 8-week-old WKY rats (WKY8w) group;

n = 7 in each group

### Vascular changes in WT%, WA%, and CA% in the thoracic aorta of SHRs, and the correlation between BP and these variables

**Fig 2:**
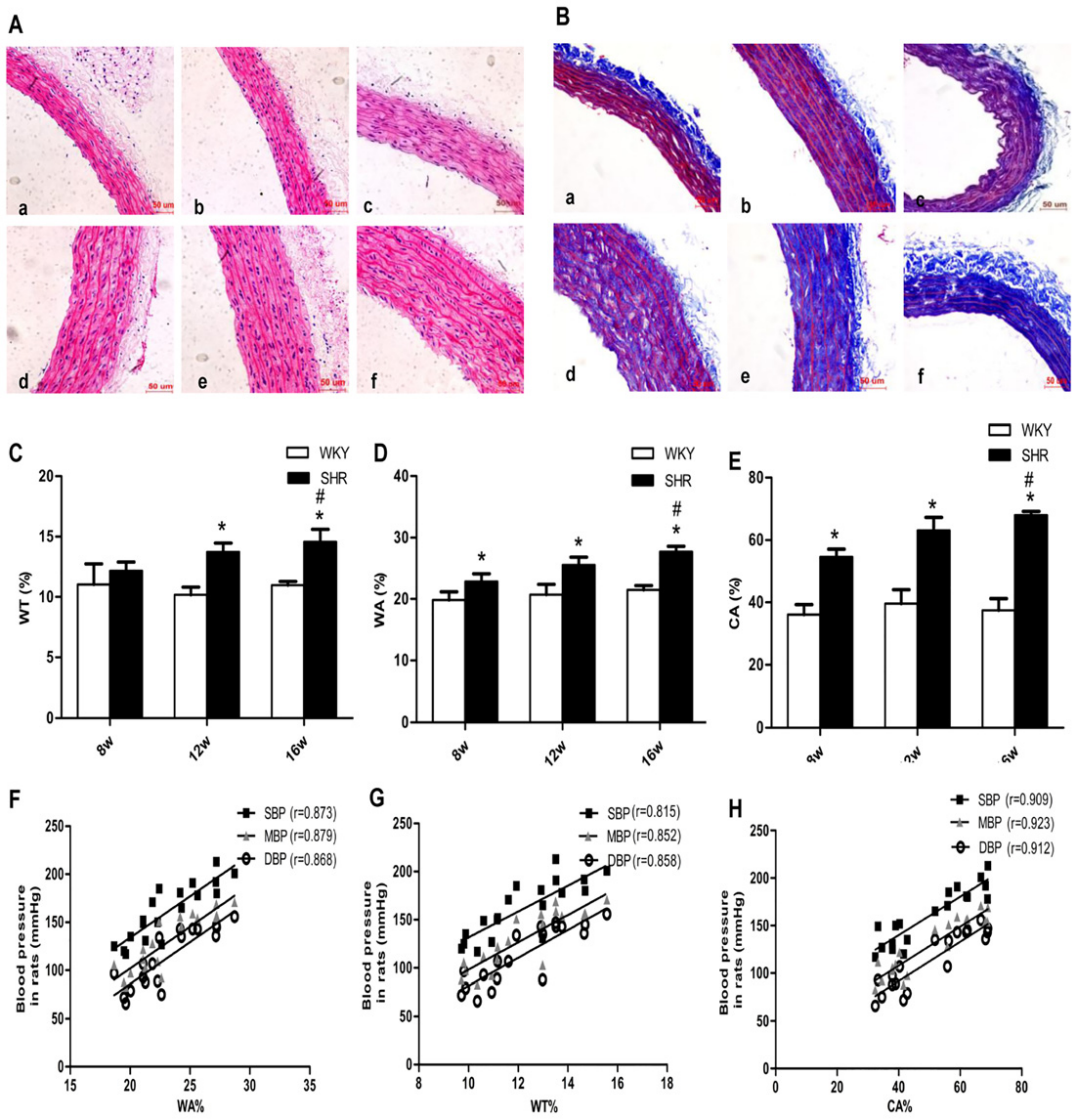
Changes related to proliferation and remodeling indices of vascular smooth muscle layer of rat thoracic aorta, and linear correlation analyses between BP and these variables. **a** Representative photomicrographs showing hematoxylin and eosin staining (200 ×). **b** Representative photomicrographs showing Masson trichrome staining (200 ×). Masson trichrome-stained collagen appears blue in color. a Eight-week-old Wistar-Kyoto rat group (WKY8w). b Twelve-week-old Wistar-Kyoto rat group (WKY12w). c Sixteen-week-old Wistar-Kyoto rat group (WKY16w). d SHR8w. **e** Twelve-week-old SHRs group (SHR12w). **f**SHR16w. **c-e** Comparison of the wall thickness percentage of the external diameter (WT%) (**c**) Total vessel wall area as a percentage of the total area (WA%) (**d**) Total area occupied by collagen as a percentage of the total vessel wall area (CA%) (**e**) in different groups using Image-Pro Plus 6.0 software. **f-h** Analyses of correlation between BP and WA% (**f**) WT% (**g**) CA% (**h**), respectively, using Image-Pro Plus 6.0 software. Data are presented as means ± standard deviation. *P < 0.05 SHRs groups versus the age-matched WKY groups; ^#^*P* < 0.05 SHR16w group versus SHR8w group; n = 7 in each group. *P* < 0.05 for each correlation coefficient (n = 7).

HE and Masson trichrome staining revealed that vessel walls of thoracic arterioles were thicker (Fig 2A-(d–f) and 2B-(d–f) in the SHRs groups than in age-matched WKY rat groups (Fig 2A-(a–c) and 2B-(a–c)). In addition, Masson trichrome staining (Fig 2B) indicated a high degree of collagen deposition localized to the media of the thoracic arteriole in the SHRs groups. Correspondingly, the WA%, WT%, and CA% of thoracic aortas were significantly increased in the SHRs groups or in SHR16w group compared with the age-matched WKY rat groups or SHR8w group, respectively (*P* < 0.05). There was no difference between the WKY rat groups (*P* > 0.05) (Fig 2C–2E). Linear regression analysis showed that WA%, WT%, and CA% were positively correlated with BP in rats (WA%: r = 0.873 for SBP, r = 0.868 for DBP, r = 0.879 for MAP; WT%: r = 0.815 for SBP, r = 0.858 for DBP, r = 0.852 for MAP; CA%: r = 0.909 for SBP, r = 0.912 for DBP, r = 0.923 for MAP; *P*< 0.05, respectively) (Fig 2F–2H).

### Expression of proliferation and remodeling marker proteins in the thoracic aorta of SHRs

**Fig 3:**
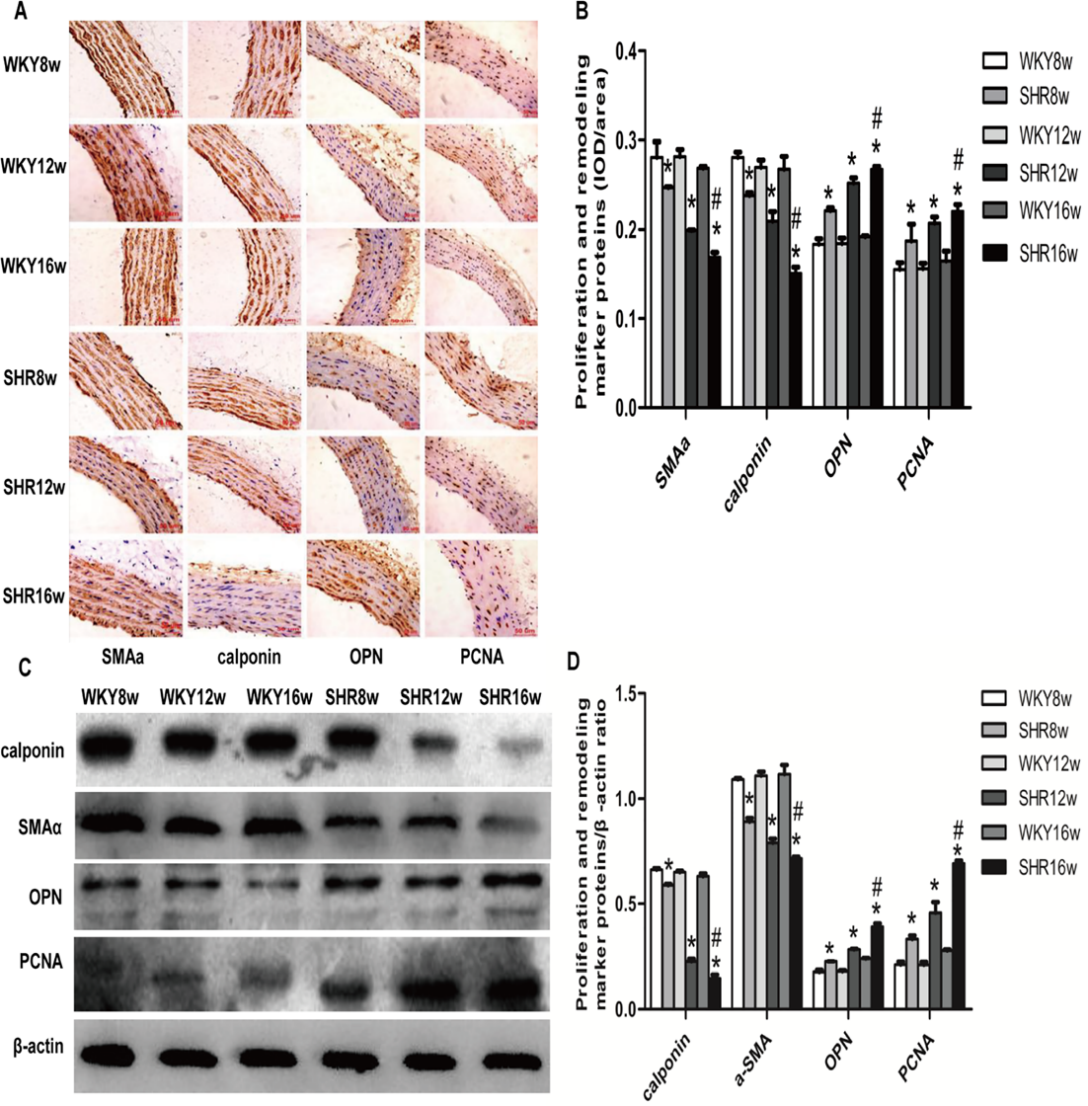
Expression of proliferation and remodeling marker proteins in the thoracic aorta of rats. **a** Immunohistochemical detection of proliferation and remodeling marker proteins including smooth muscle a-actin (SMAα), calponin, osteopontin (OPN), and proliferating cell nuclear antigen (PCNA) in the media of thoracic aorta in rats (200 ×). **b** Densitometric analysis of (**a**) using Image-Pro Plus 6.0 software. Data are presented as means ± standard deviation.*P < 0.05 SHRs groups versus the age-matched WKY groups; ^#^*P* < 0.05 SHR16w group versus SHR8w group; n = 7 in each group. **c** Proliferation and remodeling marker proteins in thoracic aorta of rats detected by western blotting. **d** Densitometric analysis of (c). Data are presented as means ± standard deviation.*P < 0.05 SHRs groups versus the age-matched WKY groups; ^#^*P* < 0.05 SHR16w group versus SHR8w group; n = 7 in each group.

In order to further examine the changes in vascular proliferation and remodeling due to hypertension, we performed Immunohistochemical analysis (see S2 Table for data) (Fig 3A) and western blotting (see S3 Table for data) (Fig 3C) of rat thoracic aortas. Our results showed that the expression of OPN, a synthetic/dedifferentiated phenotype marker protein, and PCNA was markedly increased, whereas the expression of SMAα and calponin, two contractile/differentiated phenotype marker proteins was significantly decreased in the media of thoracic aorta in SHR groups in comparison with the age-matched WKY rat groups (*P* < 0.05). The same trends were observed on comparing the SHR16w group with the SHR8w group (*P* < 0.05) (Fig 3B and 3D).

### CaSR expression in the thoracic aorta of SHRs and CaSR protein levels in SHRs and plasma from hypertensive patients

**Fig 4:**
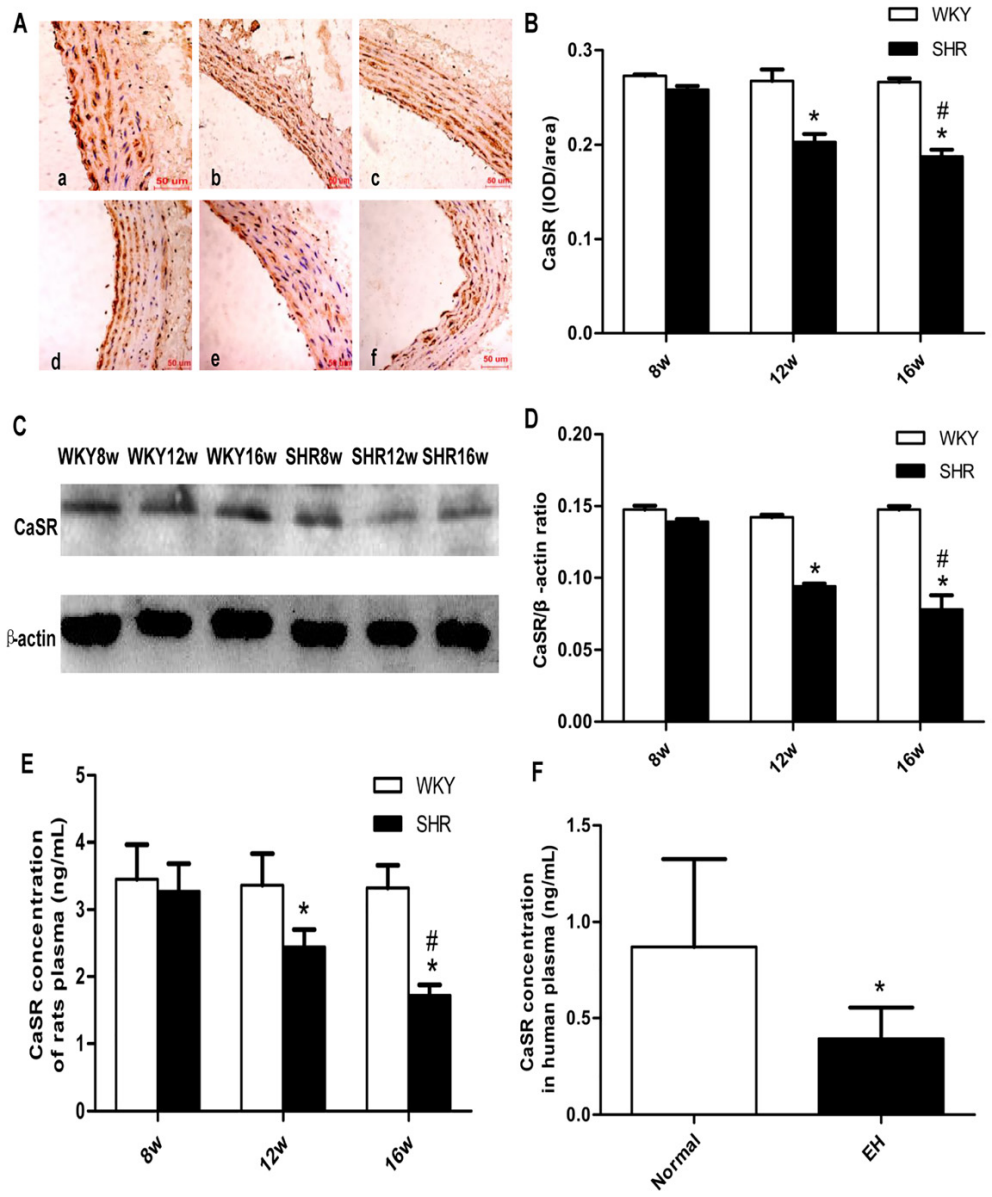
Expression and protein level of the extracellular calcium-sensing receptor (CaSR) were decreased in the hypertension groups. **a** Immunohistochemical detection of CaSR in thoracic aorta of rats (200 ×): WKY8w (a),WKY12w (b), WKY16w (c), SHR8w (d), SHR12w (e), SHR16w (f) **b** Densitometric analysis of (**a**) using Image-Pro Plus 6.0 software; data are presented as means ± standard deviation.*P < 0.05 SHRs groups versus the age-matched WKY groups; ^#^*P* < 0.05 SHR16w group versus SHR8w group; n = 7 in each group **c** CaSR expression in thoracic aorta of rats detected by western blotting. **d** Densitometric analysis of (**c**) using Image-Pro Plus 6.0 software. Data are presented as means ± standard deviation.*P < 0.05 SHRs groups versus the age-matched WKY groups; ^#^*P* < 0.05 SHR16w group versus SHR8w group; n = 7 in each group. **e** CaSR level in rat plasma (as detected by ELISA). Data are presented as means ± standard.*P < 0.05 SHRs groups versus the age-matched WKY groups; ^#^*P* < 0.05 SHR16w group versus SHR8w group; n = 7 in each group. **f** CaSR content in human plasma (as detected by ELISA). Data are presented as means ± standard deviation;*P < 0.05 EH group versus normal group (n = 100).

Immunohistochemistry (see S4 Table for data) (Fig 4A) and western blotting analysis (see S5 Table for data) (Fig 4C) of rat thoracic aorta showed that the expression of CaSR was lower in the SHR groups than in the age-matched WKY rat groups (*P* < 0.05), except for the 8-week-old rat group. The same trends were observed on comparing the SHR16w group with the SHR8w group (*P* < 0.05) (Fig 4B and 4D). The CaSR content in the plasma of rats, as detected by ELISA, (see S6 Table for data) were also consistent with the above (*P* < 0.05) (Fig 4E). In the human groups, the CaSR content in the plasma samples from the hypertension group was significantly lower than in the normal group (*P* < 0.05) (Fig 4F).

### Levels of cAMP, renin, and Ang II in the plasma of SHRs and hypertensive patients, and the correlations between BP and these variables

**Fig 5:**
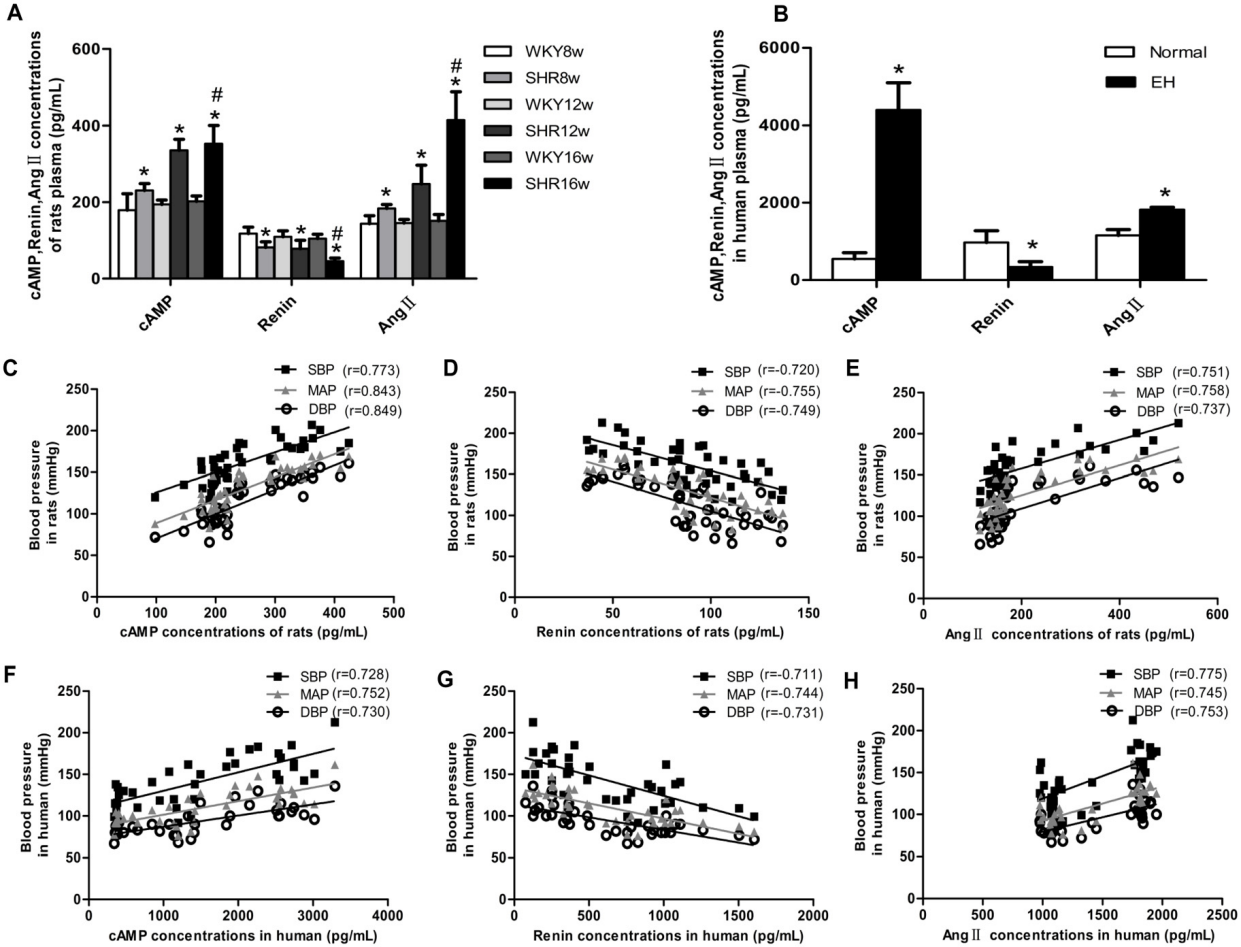
Levels of cAMP, renin, and Ang II in human and rat plasma, and analyses of linear correlation between BP and these variables. **a** Levels of cAMP, renin, and Ang II in rat plasma (detected by ELISA). Data are presented as means ± standard deviation.*P < 0.05 SHRs groups versus the age-matched WKY groups; ^#^*P* < 0.05 SHR16w group versus SHR8w group; n = 7 in each group. **b** Levels of cAMP, renin, and Ang II in human plasma (detected by ELISA). Data are presented as means ± standard deviation.*P < 0.05, EH group versus normal group. **c-e** Analysis of linear correlation between cAMP (**c**), renin (**d**), Ang II (**e**) content in rat plasma (detected by ELISA) and BP. *P* < 0.05 for each correlation coefficient (n = 7) **f-h** Analysis of linear correlation between cAMP (**f**), renin (**g**), Ang II (**h**) content in human plasma (detected by ELISA) and BP. *P* < 0.05 for each correlation coefficient (n = 100).

Analysis of rat plasma by ELISA revealed that the level of cAMP and Ang II were higher in the SHRs groups than in the age-matched WKY rat groups(see S7 Table for data) (*P* < 0.05) (Fig 5A). The same trends were observed when the SHR16w group was compared with the SHR8w group (*P* < 0.05). However, the renin content of the rat plasma exhibited the opposite trend (Fig 5A); the renin content in the SHR groups was lower than in the age-matched WKY rats groups and the renin content in the plasma in SHR16w group was lower than those in SHR8w group. Similar trends were observed in the human plasma samples(see S8 Table for data) (*P* < 0.05) (Fig 5B). The above data showed that, with the increase of BP, the contents of cAMP and Ang II increased, whereas the content of renin in the plasma decreased. The correlation between BP and these variables was also confirmed by linear regression analyses, which showed that BP was positively correlated with cAMP and Ang II in rats (cAMP: r = 0.773 for SBP, r = 0.849 for DBP, r = 0.843 for MAP; Ang II: r = 0.751 for SBP, r = 0.737 for DBP, r = 0.758 for MAP; *P* < 0.05, Fig 5C and 5E) and human plasma samples (cAMP: r = 0.728 for SBP, r = 0.730 for DBP, r = 0.752 for MAP; Ang II: r = 0.775 for SBP, r = 0.753 for DBP, r = 0.745 for MAP; *P* < 0.05, Fig 5F and 5H), and negatively correlated with renin content (rats: r = -0.720 for SBP, r = -0.749 for DBP, r = -0.755 for MAP; human: r = -0.711 for SBP, r = -0.731 for DBP, r = -0.744 for MAP; *P* < 0.05, Fig 5D and 5G)

### Correlation between CaSR and BP as well as other variables

**Fig 6:**
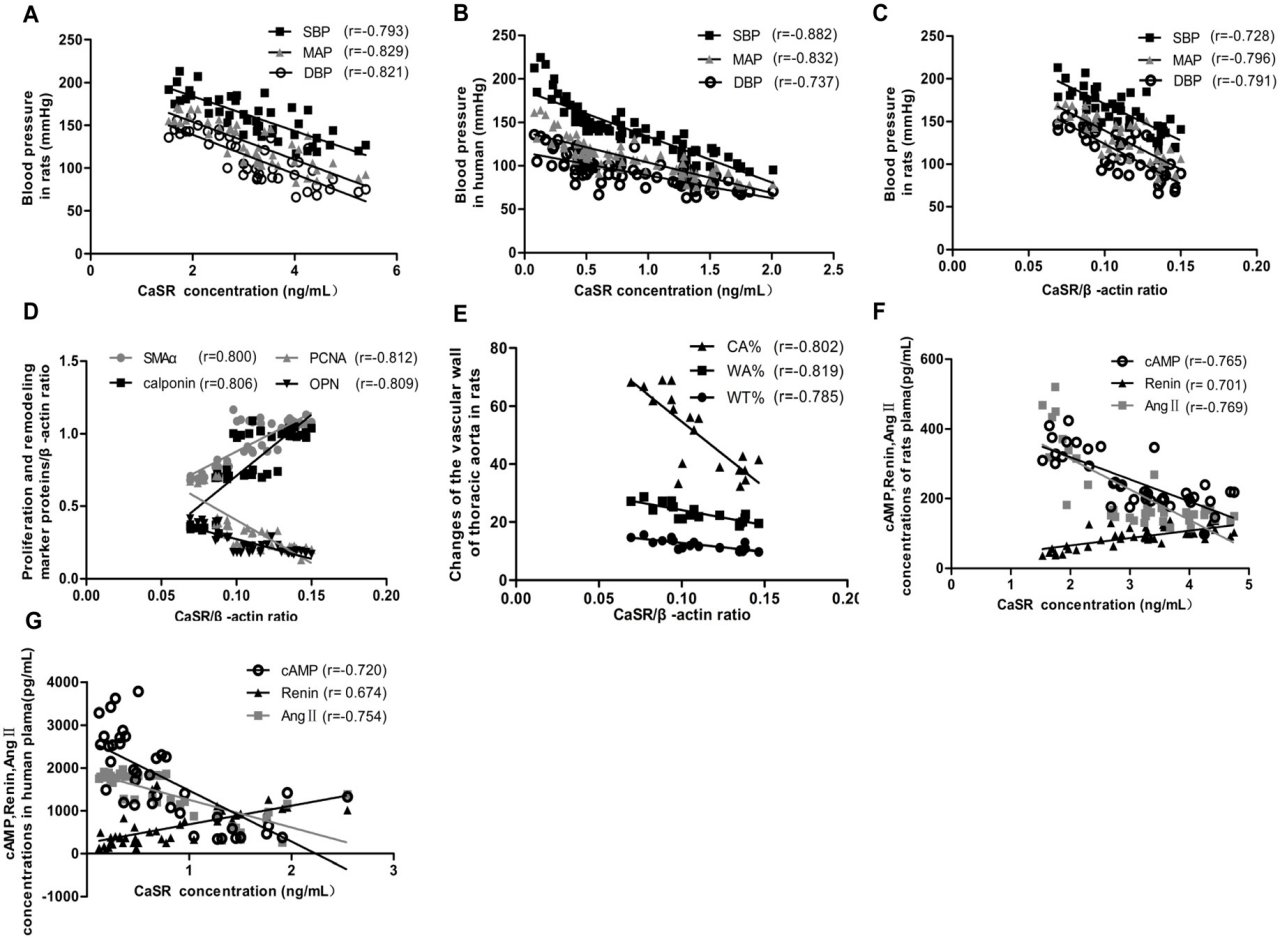
Analysis of linear correlation between CaSR expression and BP, proliferation and remodeling marker proteins, as well as cAMP, renin, and Ang II. **a** Analysis of linear correlation between CaSR content in rat plasma (detected by ELISA) and BP. **b** Analysis of linear correlation between CaSR content in human plasma (detected by ELISA) and BP. *P* < 0.05 for each correlation coefficient (n = 40). **c** Analysis of linear correlation between CaSR expression in thoracic aorta of rats (detected by western blotting) and BP. **d** Analysis of linear correlation between CaSR expression and proliferation and remodeling marker proteins in thoracic aorta of rats (detected by western blotting). **e** Analysis of linear correlation between CaSR expression in thoracic aorta of rats detected by western blotting and WT%, WA %, and CA%. **f** Analysis of linear correlation between the content of CaSR and that of cAMP, renin, and Ang II in rat plasma (detected by ELISA). *P* < 0.05 for each correlation coefficient (n = 7).**g** Analysis of linear correlation between the content of CaSR and that of cAMP, renin, and Ang II in human plasma (detected by ELISA). *P* < 0.05 for each correlation coefficient (n = 100).

In order to further explore the correlation between CaSR and BP, linear correlation analysis was performed. The results showed that the CaSR content in rat plasma (r = -0.793 for SBP, r = -0.821 for DBP r = -0.829 for MAP, *P* < 0.05) and CaSR expression in the thoracic aortas (r = -0.728 for SBP, r = -0.791 for DBP, r = -0.796 for MAP, *P* < 0.05) decreased gradually with an increase in BP (Fig 6A and 6C). Moreover, this surprising negative correlation between CaSR content and BP was also observed in human plasma samples (r = -0.882 for SBP, r = -0.737 for DBP, r = -0.832 for MAP, *P* < 0.05, Fig 6B).

According to the results of Western blot analysis, we performed the correlation analyses between CaSR and the proliferation and remodeling marker proteins (Fig 6D). The results showed that significant negative correlations were observed between CaSR and OPN as well as PCNA (r = -0.809, r = -0.812, *P* < 0.05, respectively), whereas significant positive correlations were present between CaSR and SMAα as well as calponin (r = 0.800, r = 0.806, *P* < 0.05, respectively). In addition, we found linear negative correlations between CaSR and WA%, WT%, and CA% (r = -0.819, r = -0.785, r = -0.802, *P* < 0.05, respectively, Fig 6E).

In addition, the relationships between CaSR and cAMP and RAS in rats and human plasma samples (Fig 6F and 6G) indicated that the expression of CaSR was positively related to renin content (r = 0.701 for rats, r = 0.674 for humans, *P* < 0.05) and negatively correlated with cAMP and Ang II(r = -0.765 and r = -0.769 for rats, r = -0.720 and r = -0.754 for humans, *P* < 0.05).

### Correlation between the levels of the proliferation and remodeling marker proteins and BP as well as cAMP and RAS

**Fig 7:**
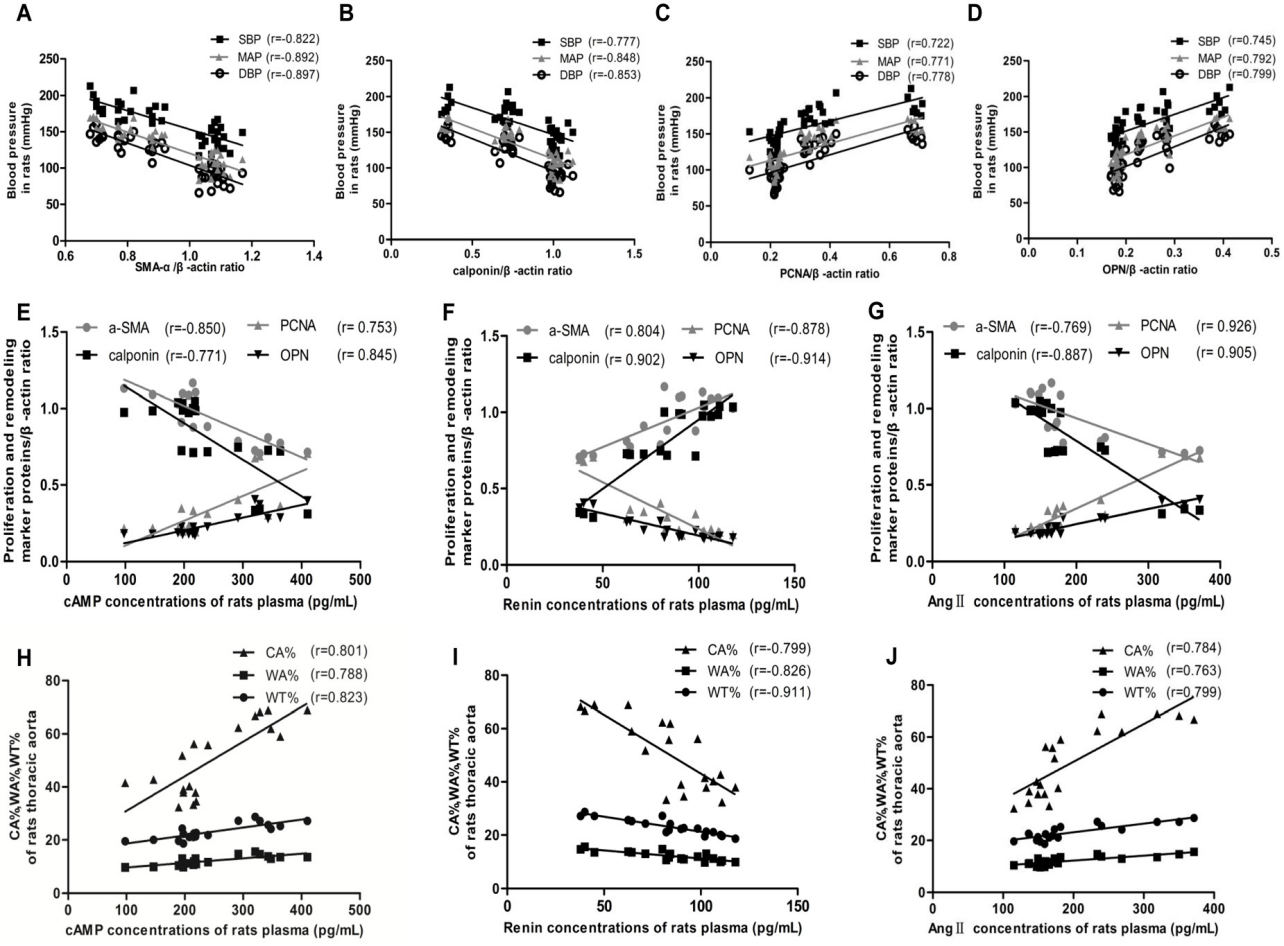
Analysis of linear correlation between proliferation- and remodeling-related indicators and BP, as well as cAMP, renin, and Ang II. **a** Analysis of linear correlation between SMAα expression in thoracic aorta of rats (detected by western blotting) and BP. **b** Analysis of linear correlation between calponin expression in thoracic aorta of rats (detected by western blotting) and BP. **c** Analysis of linear correlation between PCNA expression in thoracic aorta of rats (detected by western blotting) and BP. **d** Analysis of linear correlation between OPN expression in thoracic aorta of rats (detected by western blotting) and BP. **e-g** Analysis of linear correlation between the expression of proliferation and remodeling marker proteins in thoracic aorta of rats (detected by western blotting) and concentrations of cAMP (**e**), renin (**f**), and Ang II(**g**) in rat plasma (detected by ELISA). *P* < 0.05 for each correlation coefficient (n = 7).**h-j** Analysis of linear correlation between CA%, WA%, WT% in thoracic aorta of rats and cAMP (**h**), renin (**i**), and Ang II(**j**) concentrations in rat plasma (detected by ELISA). CA%, WA%, and WT% were measured using Image-Pro Plus 6.0 software. *P* < 0.05 for each correlation coefficient (n = 7).

In addition, we performed linear correlation analyses between BP and the levels of the proliferation and remodeling marker proteins(Fig 7), and found that BP was positively correlated with the levels of PCNA(r = 0.722 for SBP, r = 0.778 for DBP, r = 0.771 for MAP, *P* < 0.05, Fig 7C) and OPN (r = 0.745 for SBP, r = 0.799 for DBP, r = 0.792 for MAP, *P* < 0.05, Fig 7D), and negatively correlated with the level of SMAα (r = -0.822 for SBP, r = -0.897 for DBP, r = -0.892 for MAP, *P* < 0.05, Fig 7A) and calponin (r = -0.777 for SBP, r = -0.853 for DBP, r = -0.848 for MAP, *P* < 0.05, Fig 7B). The correlation coefficient between DBP and the level of these proteins was found to be the highest.

Taken together, the results of the analysis of linear correlation between cAMP and RAS in rat plasma and the proliferation and remodeling marker proteins, as well as WA%, WT%, and CA% (Fig 7) showed that the level of renin is negatively correlated with that of PCNA and OPN (r = -0.878 and r = -0.914, *P* < 0.05, Fig 7F) and WA%, WT%, and CA% (r = -0.826, r = -0.911, and r = -0.799, *P* < 0.05, Fig 7I), and positively correlated with the levels of SMAα and calponin (r = 0.804, r = 0.902, *P* < 0.05, respectively, Fig 7F). Conversely, cAMP, Ang II exhibited positive correlation with PCNA and OPN (r = 0.753 and r = 0.845 for cAMP; r = 0.926 and r = 0.905 for Ang II; *P* < 0.05, Fig 7E and 7G), WA%, WT%, and CA% (r = 0.788, r = 0.823, and r = 0.801 for cAMP; r = 0.763, r = 0.799 and r = 0.784 for Ang II; *P* < 0.05, Fig 7H and 7J); and negative correlation with SMAα and calponin levels (r = -0.850 and r = -0.771 for cAMP; r = -0.769 and r = -0.887 for Ang II; *P* < 0.05, Fig 7E and 7G)

## Discussion

The results of the current study show that increased BP is associated with decreased CaSR expression in the SHR model as well as in the plasma of humans. We additionally found that, concomitant with the decrease in the expression of CaSR, the level of renin decreased whereas that of cAMP and Ang II increased. The RAS plays an important role in the regulation of BP[24]. Therefore, we hypothesized that a decrease in the expression of CaSR may contribute to hypertension, possibly though activation of the RAS. To our knowledge, this is the first study exploring the correlation between the CaSR and EH and the mechanisms underlying this association.

Hypertension leads to phenotypic changes in VSMCs in the vascular wall. The remodeling of large arteries due to such changes in VSMCs contributes to the development of hypertension, and associated complications[25]. In the current study, we observed that the expression of SMAα and calponin(two contractile/differentiated phenotype marker proteins[26]) was decreased, whereas that of OPN(a synthetic/dedifferentiated phenotype marker protein[26]) and PCNA (an important index of cell proliferation[27]) was markedly increased within the thoracic aorta in rats. Consistent with these findings, the WA%, WT%, and CA% of the thoracic aorta were increased significantly in SHRs. In addition, the levels of OPN and PCNA, WA%, WT%, and CA% correlated positively with BP, which indicated that hypertension caused by cell proliferation and remodeling in the thoracic aorta resulted from an increase in arterial stiffness and decrease in vascular elasticity. The correlation between diastolic BP and the levels of OPN and PCNA was stronger than that between systolic BP and these proteins, indicating the predominant effect of wall elasticity on diastolic BP.

The relationship between the CaSR and EH suggests that CaSR agonists may lower BP[10]. The role of the CaSR in the regulation of BP may involve the RAS [15], although the specific underlying mechanism is unknown. Smajilovic et al.[28] confirmed that expression of the CaSR in rat aortic VSMCs affected their proliferation. In the current study, we hypothesized that reduced CaSR expression caused EH by promoting the phenotypic modulation of cells in the thoracic aorta. Consistent with this speculation, our study showed that the expression of the CaSR in rat thoracic aortas, as well as CaSR protein content in human and rat plasma, decreased with increasing BP. Decreased levels of the CaSR were additionally associated with increased levels of synthetic/dedifferentiated phenotype marker proteins. These changes promote thoracic artery cell proliferation and remodeling, decreasing vascular elasticity and inducing hypertension. Our results therefore suggest that the CaSR plays an important role in cell proliferation and remodeling of thoracic aorta in the SHR model.

In order to investigate the mechanism by which the CaSR affects vascular cell proliferation and remodeling in hypertension, we focused on the roles of cAMP and the RAS. The RAS is present throughout the body; approximately 15% is involved in blood circulation and 85% in the kidney, blood vessels, heart, and other tissues. The RAS mediates the contraction and relaxation of vascular smooth muscle, vascular remodeling, and the proliferation of cardiac muscle and extracellular matrix (ECM). These factors play important roles in the development of hypertension and related complications such as atherosclerosis and stroke. In the current study, it was found that levels of the CaSR in human and rat plasma, as well as CaSR expression in the rat thoracic aorta, were negatively correlated with the levels of BP and positively correlated with those of cAMP, Ang II, and renin. Our results additionally demonstrate that renin levels were negatively correlated with BP, PCNA and OPN levels, WA%, WT%, and CA%, and positively correlated with SMAα and calponin levels. Conversely, cAMP and Ang II were positively correlated with BP, PCNA and OPN levels, WA%, WT%, and CA%, and negatively correlated with SMAα and calponin concentrations. Overall, these data show that a reduction in the expression of the CaSR is correlated with vascular proliferation and remodeling in EH via activation of the RAS pathway.

We found significant negative correlation between the expression of CaSR and levels of cAMP and Ang II; however, a significant positive correlation was observed between CaSR expression and renin levels. This implies that the CaSR plays an important role in LRH. However, IP3 and intracellular Ca^2+^ are important components of the post-receptor pathway of CaSR-mediated activation[19], which suggests that the expression of CaSR influences the release of cAMP and the RAS, thereby regulating BP via the G-PLC-IP3-Ca^2+^ signal transduction pathway during the pathogenesis of hypertension. In the current study, we observed correlations between the CaSR and EH, as well between CaSR and cAMP and RAS. Further studies using CaSR agonist- and antagonist-based strategies are needed in order to elucidate the role of the CaSR in the pathogenesis of hypertension, and to understand the underlying mechanisms such as the G-PLC-IP3-Ca^2+^ signal transduction pathway.

In summary, the current study presents data demonstrating that a reduction in the expression of the CaSR is correlated with increased VSMC proliferation and remodeling, which is implicated in the development of EH via the activation of the cAMP-RAS pathway. Our findings provide new insights into the pathogenesis of this complex disease, and suggest that the vascular CaSR pathway may be a therapeutic target in the management of hypertension.

## Supporting Information

S1 Table Comparison of blood pressure in rats. (DOCX)

S2 Table The Immunohistochemical detection of proliferation and remodeling marker proteins in the thoracic aorta of rats in each group. (DOCX)

S3 Table The relative expression of proliferating remodeling protein in the thoracic aorta of rats in each group. (DOCX)

S4 Table Immunohistochemical detection of CaSR in thoracic aorta of rats in each group. (DOCX)

S5 Table CaSR relative expression in thoracic aorta of rats detected by western blotting. (DOCX)

S6 Table CaSR levels in rat and human plasma detected by ELISA. (DOCX)

S7 Table The concentrations of cAMP, renin, and Ang II in the plasma of rats. (DOCX)

S8 Table The level of cAMP, renin, and Ang II in the plasma of human. (DOCX)
