# Supplementary material for: Reduced Expression of the Extracellular Calcium-Sensing Receptor (CaSR) Is Associated with Activation of the Renin-Angiotensin System (RAS) to Promote Vascular Remodeling in the Pathogenesis of Essential Hypertension
Source: PLoS One. 2016 Jul 8;11(7):e0157456. doi: 10.1371/journal.pone.0157456 (PMC4938397; doi:10.1371/journal.pone.0157456)
Supplement: S1 Table — (DOCX) [file pone.0157456.s001.docx]

S1 Table Comparison of blood pressure between hypertension and normal blood pressure group in

human. ($\bar{x}$±S，n=100)

**P* < 0.05 Hypertension group versus normal blood group.

| Groups | SBP  (mmHg) | DBP  (mmHg) | MAP  (mmHg) |
| --- | --- | --- | --- |
| Normal | 123.94±22.60 | 84.19±14.58 | 97.44±16.68 |
| Hypertension | 163.22±22.92* | 103.63±15.83* | 123.49±16.99* |
