# Supplementary material for: Reduced Expression of the Extracellular Calcium-Sensing Receptor (CaSR) Is Associated with Activation of the Renin-Angiotensin System (RAS) to Promote Vascular Remodeling in the Pathogenesis of Essential Hypertension
Source: PLoS One. 2016 Jul 8;11(7):e0157456. doi: 10.1371/journal.pone.0157456 (PMC4938397; doi:10.1371/journal.pone.0157456)
Supplement: S2 Table — (DOCX) [file pone.0157456.s002.docx]

S2 Table The Immunohistochemical detection of proliferation and remodeling marker proteins in the thoracic aorta of rats in each group

| Groups | SMAa(IOD/area) | calponin(IOD/area) | OPN(IOD/area) | PCNA(IOD/area) |
| --- | --- | --- | --- | --- |
| WKY8w | 0.281±0.018 | 0.281±0.006 | 0.184±0.006 | 0.156±0.007 |
| SHR8w | 0.247±0.001* | 0.238±0.003* | 0.221±0.003* | 0.187±0.019* |
| WKY12w | 0.282±0.008 | 0.270±0.008 | 0.184±0.006 | 0.156±0.006 |
| SHR12w | 0.120±0.001* | 0.210±0.010* | 0.252±0.006* | 0.207±0.007* |
| WKY16w | 0.269±0.002 | 0.268±0.014 | 0.192±0.002 | 0.165±0.011 |
| SHR16w | 0.169±0.005*^,#^ | 0.151±0.007*^,#^ | 0.267±0.003*^,#^ | 0.221±0.007*^,#^ |

**P* < 0.05 SHRs groups versus the age-matched WKY groups; ^#^*P* < 0.05 SHR16w group versus SHR8w group.
