# Supplementary material for: Reduced Expression of the Extracellular Calcium-Sensing Receptor (CaSR) Is Associated with Activation of the Renin-Angiotensin System (RAS) to Promote Vascular Remodeling in the Pathogenesis of Essential Hypertension
Source: PLoS One. 2016 Jul 8;11(7):e0157456. doi: 10.1371/journal.pone.0157456 (PMC4938397; doi:10.1371/journal.pone.0157456)
Supplement: S3 Table — (DOCX) [file pone.0157456.s003.docx]

S3 Table The relative expression of proliferating remodeling protein in the thoracic aorta of rats in each group($\bar{x}$±S，n=7)

| Groups | Calponin/β-actin ratio | α-SMA/β-actin ratio | OPN/β-actin  ratio | PCNA/β-actin  ratio |
| --- | --- | --- | --- | --- |
| WKY8w | 0.662±0.006 | 1.092±0.006 | 0.177±0.009 | 0.211±0.013 |
| SHR8w | 0.588±0.006* | 0.891±0.018* | 0.225±0.001* | 0.332±0.017* |
| WKY12w | 0.651±0.006 | 1.110±0.020 | 0.181±0.004 | 0.209±0.014 |
| SHR12w | 0.225±0.013* | 0.791±0.018* | 0.283±0.003* | 0.457±0.051* |
| WKY16w | 0.631±0.014 | 1.117±0.043 | 0.240±0.002 | 0.277±0.006 |
| SHR16w | 0.147±0.017*^,#^ | 0.716±0.009*^,#^ | 0.391±0.017*^,#^ | 0.692±0.014*^,#^ |

Data are presented as means ± standard deviation. **P* < 0.05 SHRs groups versus the age-matched WKY groups;
