# Supplementary material for: Reduced Expression of the Extracellular Calcium-Sensing Receptor (CaSR) Is Associated with Activation of the Renin-Angiotensin System (RAS) to Promote Vascular Remodeling in the Pathogenesis of Essential Hypertension
Source: PLoS One. 2016 Jul 8;11(7):e0157456. doi: 10.1371/journal.pone.0157456 (PMC4938397; doi:10.1371/journal.pone.0157456)
Supplement: S4 Table — (DOCX) [file pone.0157456.s004.docx]

S4 Table Immunohistochemical detection of CaSR in thoracic aorta of rats in each group(200 ×) ($\bar{x}$±S，n=7)

| Groups | CaSR (IOD/area) |
| --- | --- |
| WKY8w | 0.273±0.002 |
| SHR8w | 0.259±0.004 |
| WKY12w | 0.267±0.012 |
| SHR12w | 0.203±0.009* |
| WKY16w | 0.266±0.004 |
| SHR16w | 0.188±0.007*^,#^ |

**P* < 0.05 SHRs groups versus the age-matched WKY groups; ^#^*P* < 0.05 SHR16w group versus SHR8w group.
