# Supplementary material for: Reduced Expression of the Extracellular Calcium-Sensing Receptor (CaSR) Is Associated with Activation of the Renin-Angiotensin System (RAS) to Promote Vascular Remodeling in the Pathogenesis of Essential Hypertension
Source: PLoS One. 2016 Jul 8;11(7):e0157456. doi: 10.1371/journal.pone.0157456 (PMC4938397; doi:10.1371/journal.pone.0157456)
Supplement: S5 Table — (DOCX) [file pone.0157456.s005.docx]

| S5 Table CaSR relative expression in thoracic aorta of rats detected by western blotting($\bar{x}$±S，n=7) | |
| --- | --- |
| Groups | CaSR/β-actin ratio |
| WKY8w | 0.139±0.002 |
| SHR8w | 0.148±0.003 |
| WKY12w | 0.142±0.002 |
| SHR12w | 0.094±0.001* |
| WKY16w | 0.148±0.003 |
| SHR16w | 0.078±0.010*^,#^ |

**P* < 0.05 SHRs groups versus the age-matched WKY groups; ^#^*P* < 0.05 SHR16w group versus SHR8w group;
