# Supplementary material for: Reduced Expression of the Extracellular Calcium-Sensing Receptor (CaSR) Is Associated with Activation of the Renin-Angiotensin System (RAS) to Promote Vascular Remodeling in the Pathogenesis of Essential Hypertension
Source: PLoS One. 2016 Jul 8;11(7):e0157456. doi: 10.1371/journal.pone.0157456 (PMC4938397; doi:10.1371/journal.pone.0157456)
Supplement: S6 Table — (DOCX) [file pone.0157456.s006.docx]

| S6 Table CaSR levels in rat and human plasma detected by ELISA($\bar{x}$±S，n=7) | |
| --- | --- |
| Groups | CaSR concentration in rats and human plasma (ng/mL) |
| WKY8w | 3.452±0.817 |
| SHR8w | 3.275±0.610 |
| WKY12w | 3.366±0.471 |
| SHR12w | 2.443±0.457* |
| WKY16w | 3.321±0.339 |
| SHR16w | 1.720±0.157*^,#^ |
| Normal | 1.193±0.508 |
| Hypertension | 0.547±0.218^a^ |

**P* < 0.05 SHRs groups versus the age-matched WKY groups; ^#^*P* < 0.05 SHR16w group versus SHR8w group; ^a^ *P*<0.05 hypertension group versus normal blood pressure group.
