# Supplementary material for: Reduced Expression of the Extracellular Calcium-Sensing Receptor (CaSR) Is Associated with Activation of the Renin-Angiotensin System (RAS) to Promote Vascular Remodeling in the Pathogenesis of Essential Hypertension
Source: PLoS One. 2016 Jul 8;11(7):e0157456. doi: 10.1371/journal.pone.0157456 (PMC4938397; doi:10.1371/journal.pone.0157456)
Supplement: S7 Table — (DOCX) [file pone.0157456.s007.docx]

S7 Table The concentrations of cAMP, renin, and Ang II in the plasma of rats($\bar{x}$±S，n=7)

| Groups | cAMP(pg /mL) | Renin(pg /mL) | AngⅡ(pg /mL) |
| --- | --- | --- | --- |
| WKY8w | 179.270±42.932 | 117.725±17.115 | 143.805±21.048 |
| SHR8w | 230.826±17.927* | 81.814±14.285* | 183.584±10.224* |
| WKY12w | 194.074±11.775 | 109.833±15.359 | 145.259±9.287 |
| SHR12w | 335.742±29.256* | 78.433±22.008* | 247.887±49.071* |
| WKY16w | 202.446±13.786 | 104.971±11.252 | 151.307±16.343 |
| SHR16w | 352.704±48.147*^,#^ | 45.952±7.714*^,#^ | 414.793±73.519*^,#^ |

**P* < 0.05 SHRs groups versus the age-matched WKY groups; ^#^*P* < 0.05 SHR16w group versus SHR8w group.
