# Supplementary material for: Reduced Expression of the Extracellular Calcium-Sensing Receptor (CaSR) Is Associated with Activation of the Renin-Angiotensin System (RAS) to Promote Vascular Remodeling in the Pathogenesis of Essential Hypertension
Source: PLoS One. 2016 Jul 8;11(7):e0157456. doi: 10.1371/journal.pone.0157456 (PMC4938397; doi:10.1371/journal.pone.0157456)
Supplement: S8 Table — (DOCX) [file pone.0157456.s008.docx]

S8 Table The level of cAMP, renin, and Ang II in the plasma of human($\bar{x}$±S，n=100)

| Groups | cAMP(pg/mL) | Renin(pg/mL) | AngⅡ(pg/mL) |
| --- | --- | --- | --- |
| Normal | 545.203±161.329 | 973.332±275.952 | 1157.298±148.541 |
| Hypertension | 4400.170±699.697^a^ | 335.969±140.208^a^ | 1822.270±60.161^a^ |

**P* < 0.05 Hypertension group versus normal blood group.
